# Matrix-Assisted Laser Desorption/Ionization (MALDI) Mass Spectrometry Imaging of L-4-Phenylalanineboronic Acid (BPA) in a Brain Tumor Model Rat for Boron Neutron Capture Therapy (BNCT)

**DOI:** 10.5702/massspectrometry.A0105

**Published:** 2022-12-20

**Authors:** Yumi Miyake, Sachie Kusaka, Isao Murata, Michisato Toyoda

**Affiliations:** 1 Forefront Research Center, Graduate School of Science, Osaka University, 1–1 Machikaneyama, Toyonaka, Osaka 560–0043, Japan; 2 Division of Sustainable Energy and Environmental Engineering, Graduate School of Engineering, Osaka University, aoka 2–1, Suita, Osaka 565–0871, Japan; 3 MS open innovation project in JEOL YOKOGUSHI Research Alliance Laboratories, Osaka University, 1–1 Machikaneyama, Toyonaka, Osaka 560–0043, Japan

**Keywords:** mass spectrometry imaging, matrix-assisted laser desorption/ionization (MALDI), boron neutron capture therapy (BNCT), L-4-phenylalanineboronic acid (BPA), brain tumor, melanoma

## Abstract

Boron neutron capture therapy (BNCT) is a cell-selective particle therapy for cancer using boron containing drugs. Boron compounds are accumulated in high concentration of tens ppm level of boron in target tumors to cause lethal damage to tumor tissue. The examination of boron distribution in target tumor and normal tissue is important to evaluate the efficiency of therapy. The matrix-assisted laser desorption/ionization (MALDI) mass spectrometry imaging (MSI) is a powerful tool to visualize the distribution of target analyte in biological samples. In this manuscript, we report a trial to visualize the distribution of a typical BNCT drug, L-4-phenylalanine boronic acid (BPA) in a brain tumor model rat using MALDI-MSI technique. We performed a MALDI-MSI with high mass resolution targeting to [BPA+H]^+^ at *m*/*z* 210 in a BPA-treated rat brain section using a spiral orbit-type time of flight (SpiralTOF) mass spectrometer. Several BPA ion species, [BPA+H]^+^, [BPA−H_2_O+Na]^+^, [BPA+DHB−2H_2_O+Na]^+^ and [BPA+DHB−2H_2_O+K]^+^ were detected separate from peaks originated from biomolecules or matrix reagent by achieving the mass resolving power of approximately 20,000 (full width at half maximum; FWHM) at *m*/*z* 210. The mass images with 60 μm spatial resolution obtained from these BPA ion species in a mass window of 0.02 Da revealed their localization in the tumor region. Additionally, the mass image obtained from [BPA+H]^+^ also likely showed the distribution of BPA inside the tumor. MALDI-MSI with high mass resolution targeting to [BPA+H]^+^ has a great potential to visualize the distribution of BPA in brain tissue with tumor.

## INTRODUCTION

Boron neutron capture therapy (BNCT) is a cell-selective particle therapy for cancers.^1)^ L-4-phenylalanine boronic acid (BPA) is widely used as a boron-containing drug for BNCT. BPA is delivered to the cells by the L-type amino acid transporter (LAT1). Alpha (α) and lithium-7 particles emitted *via* boron (^10^B) neutron capture reaction induced by irradiation of thermal (∼0.025 eV) or epithermal (0.5 eV ∼10 keV) neutrons, cause lethal effect to cancer cells. Boron is required to be accumulate in concentration of approximately 20–40 μg/g boron in a target tumor to damage for cancer cells.^2)^ Furthermore, the abundance ratio of boron in tumor and normal tissue (T/N ratio) is one of the important parameters to determine the effect of tumor treatment and damage for normal cells. Therefore, it is important to investigate the concentration and distribution of boron compounds in tumor and normal tissue and the time profile of uptake or excretion of boron compound in tumor tissue. Inductively coupled plasma atomic emission spectrometry (ICP-AES), inductively coupled plasma mass spectrometry (ICP-MS) are used in basic research to determine the boron concentration in tumor tissue.^3,4)^ In recent years, laser ablation-ICP-MS (LA-ICP-MS),^5)^ secondary ion mass spectrometry (SIMS)^6)^ and secondary neutral mass spectrometry (SNMS)^7)^ have been used for boron targeting imaging. However, these techniques have not been widely available yet and cannot obtain the image of biomolecules in tissue which can be interactive to boron compounds, to understand the mechanism of boron compound uptake.

Mass spectrometry imaging (MSI) enables multiple molecule-specific compound mapping and identifying molecular species in tissue section.^8,9)^ This technique has been used to visualize the localization of endogenous biomolecules such as protein, peptides,^10,11)^ lipids,^12)^ amino acids,^13)^ fatty acids,^14)^ neurotransmitters^15,16)^ and steroid hormones^17)^ as well as exogenous compounds such as pharmaceutical drugs in various tissues.^18,19)^ There are several scanning types of MSI techniques, such as matrix-assisted laser desorption/ionization (MALDI), desorption electrospray ionization (DESI),^20)^ tapping mode scanning probe electrospray ionization (t-SPESI),^21)^ SIMS,^22)^ and visualization of analyte abundance in micro-dissected tissues determined by liquid chromatography mass spectrometry (LC/MS) measurement.^23)^ MALDI-MSI has the advantages of high sensitivity and simultaneous detection of multiple organic compounds with 20–200 μm spatial resolution and enables to visualize the distribution of analytes in multiple tissues, for example tumor and normal cell region in a single image. However, biological tissues are complex mixtures of biomolecules and a great number of multiple peaks are detected. In addition, multiple peaks originated from matrix compounds are also detected in the mass range less than *m*/*z* 500 in MALDI measurements. Sugiura *et al.* reported an example of several overlapping peaks within approximately 0.1 Da around *m*/*z* 394, which is the *m*/*z* value of protonated molecule of a drug, in a liver sample in a MALDI-MSI measurement.^24)^ Therefore, peak separation is necessary to distinguish the target analyte from biomolecules or matrix compounds in biological sample. Tandem mass spectrometry (MS/MS), and use of mass spectrometer with high mass resolving power such as Fourier transform ion cyclotron resonance mass spectrometry (FT-ICRMS) are representative methods to avoid overlapping signals from isobaric compounds in tissues. The amount of administered drug distributed in tissue is usually much smaller than that of biomolecules or matrix compounds. The peaks of biomolecules or matrix compounds adjacent to target analytes not only cause the problem of overlapping, but also make it difficult to detect target analytes due to ionization suppression. MALDI-MSI technique coupled with on tissue derivatization^25,26)^ is often used to enhance the signals of analyte, which have low ionization efficiencies and also low abundance in tissue. The combination of peak separation by MS/MS or high mass resolution and derivatization technique is applied to detect the administered drug in tissue, however it is sometimes hard to perform due to low peak intensity of product ions in MS/MS measurement or low efficiency of derivatization reaction on tissue section and so on.

In contrast, the effective concentration level of boron drug in BNCT is comparable to that of lipids in brain sample to which MALDI-MSI is applied without derivatization so that it is likely to be detectable without derivatization. However, the peak separation of target analyte from biomolecules or matrix compound with high mass resolution is needed to detect BNCT drugs because most of BNCT agents are small molecules with a high probability of overlap with isobaric compounds in tissues, for example amino acids, neurotransmitters or matrix compounds. A spiral orbit-type time of flight (SpiralTOF) mass spectrometer has a 17 m flight path and the mass resolving power is more than 30,000 (full width at half maximum; FWHM) in a range of *m*/*z* 1000–2500. Satoh *et al.* reported the high mass resolution of SpiralTOF enabled to separate two peaks of phospholipid at *m*/*z* 848.556 and *m*/*z* 848.645 (0.09 Da differences) and the mass images obtained from each peak in mass window of 0.1 Da illustrated a different localization in tissue, although the mass images obtained from each peak in mass window of 0.2 Da revealed overlapped image.^27)^ Using SpiralTOF mass spectrometer, we demonstrated an effective approach of MALDI-MSI to detect BNCT drug, BPA, in a rat brain tissue with a kind of tumor, melanoma treated with BPA without derivatization or MS/MS measurement, aiming to achieve mass resolving power of approximately 20,000 (FWHM) around protonated BPA molecule at *m*/*z* 210.

## EXPERIMENTAL

### Chemicals

BPA, trifluoroacetic acid (TFA), LC/MS grade acetonitrile (ACN), polyethylene glycol (PEG) 200 and PEG600, sodium trifluoroacetate (TFANa) were purchased from FUJIFILM Wako Pure Chemical Corporation (Osaka, Japan). 2,5-Dihydroxybenzoic acid (DHB) and α-Cyano-4-hydroxycinnamic acid (CHCA) was purchased from Tokyo Chemical Industry Co., Ltd. (Tokyo, Japan).

### Brain tissue

The protocol of the experiments in this study was approved by the Animal Care and Use Committee of Osaka University (approval number: 2019-1-3). An 8 week old, 305 g, male jcl:SD rat transplanted with melanoma (B16F10) in the brain was purchased from CLEA Japan, Inc. (Tokyo, Japan). A BPA solution (10.6 mL, 20 mg/mL) was administered to a rat *via* the cervical vein for 2 h aiming to 20 μg/g or more of boron to be accumulated in melanoma described in previous study.^3)^ The brain sample of rat was immersed in isopentane chilled in liquid nitrogen for tissue frozen and stored at −80°C until use.

### Tissue sectioning

The frozen rat brain sample was sliced into 16 μm thickness using a cryostat microtome (CM1100, Leica Biosystems, Nusslosh, Germany) and mounted on a stainless steel sheet (50 μm thickness, 20 mm ×20 mm, Iwata industry, Tokyo, Japan). The brain section samples were stored at −80°C until use. The sections were brought to 25°C in a desiccator for 30 min before matrix application. Before matrix application, 0.5 μL of TFANa solution (2 mg/mL) dissolved in ACN and 1 μL of PEG200 and PEG600 mixture solution dissolved in ACN (5 mg/mL) were spotted outside the brain section on the stainless steel sheet for mass calibration, and also 100 pmol and 20 pmol of BPA standard solution was spotted the other place on the stainless steel sheet for the confirmation of detection.

### Matrix application

One microliter of 10 mg/mL of matrix DHB solution (85% ACN aqueous solution) was used for MALDI-MS measurement of BPA standard spot on a stainless steel sample plate. A matrix DHB solution dissolved in 85% ACN aqueous solution containing 0.1% TFA (v : v) was used for MSI measurement to detect the protonated BPA molecule in preference to other adduct molecules. The matrix DHB solution (1 mL, 40 mg/mL) was sprayed onto the brain section using an airbrush sprayer with 0.2 mm i.d. nozzle (Tamiya, Tokyo, Japan) and the section was dried for 10 min at 25°C. The brain section on stainless steel sheet was placed on a stainless steel sample plate using a carbon tape.

### MALDI-MS data acquisition

MALDI-MS measurement were performed using a MALDI-SpiralTOF/TOF mass spectrometer (JMS-S3000, JEOL Ltd., Akishima, Japan) with a 349 nm solid-phase laser operating at a frequency of 250 Hz. The mass spectrometer was operated with positive polarity in spiral mode and the spectra were acquired in the range of *m*/*z* 205–550 to detect the protonated BPA molecule at *m*/*z* 210 and the other BPA ion species. Aiming to secure the mass resolving power of 20,000 (FWHM) at *m*/*z* 210, the delayed extraction time (200 ns) and the other operating parameters of the mass spectrometer were optimized. An external mass calibration with PEG was performed prior to the sample measurements.

MS/MS experiments by high-energy collision-induced dissociation were performed to confirm the production of BPA-DHB complexes. Isolation of the precursor ion with a width of 1 Da was carried out using a timed ion gate and the product ions in the collision cell were further accelerated with 20 kV and detected through an offset parabolic ion mirror. The collision cell was operated in the presence of a helium gas.

The MSI scanning for the brain section was carried out at 60 μm spatial resolution (pixel size). Five spectra were accumulated in each pixel with 625 laser shots. The laser diameter was approximately 20 μm. The laser power and detector voltage were set to 42% and 58% respectively, to detect peaks of BPA ion species with detectable intensity while avoid overlapping with peaks originated from biomolecules or matrix. The regions of interest (ROI) were manually designated in tumor region or cerebral cortex region in the brain section, and the averaged spectra by a pixel were extracted from ROI using msMicroImager Extract software (ver. 3.0.0.1, JEOL Ltd., Akishima, Japan). Internal mass calibration using [DHB−H+2K]^+^ at *m*/*z* 230.946 were performed for each averaged spectrum obtained from ROI because the thickness of the brain section (16 μm) corresponds to the mass difference of 0.2 mDa in spiral mode. Another MSI measurement was also performed in the mass range *m*/*z* 20–215 to investigate the localization of matrix DHB, potassium and sodium in a brain tissue section. [DHB+K]^+^ at *m*/*z* 192.990 was used for the internal mass calibration. The mass images were extracted from averaged spectra in a mass window of 0.02 Da and visualized using msMicroImager View software (ver. 3.0.0.1, JEOL Ltd., Akishima, Japan).

## RESULTS AND DISCUSSION

### The observed BPA ion species by BPA standard measurements

**Figure 1:**
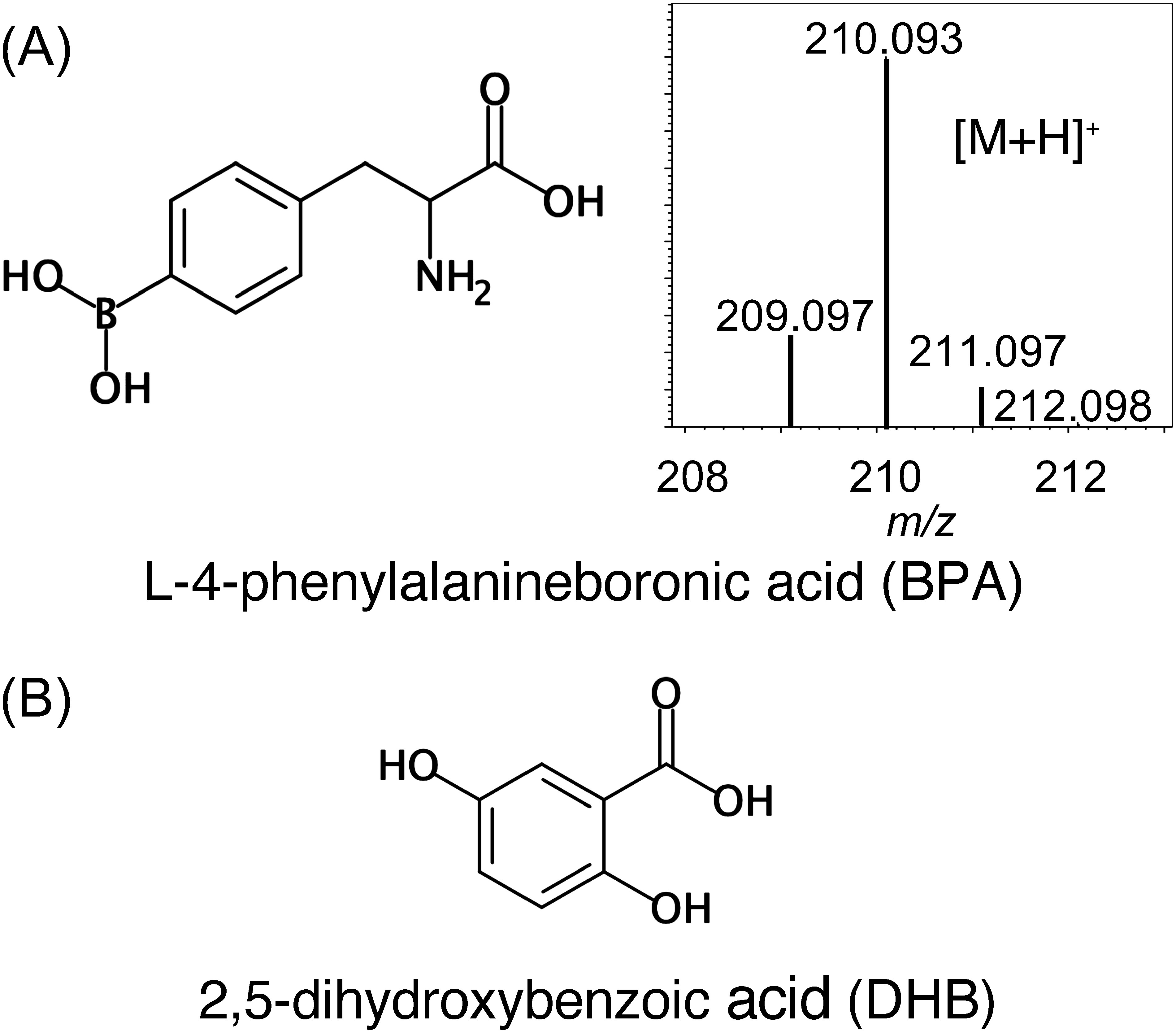
Fig. 1. (A) Chemical structure of BPA with the simulated mass spectrum and (B) chemical structure of matrix DHB.

**Figure 2:**
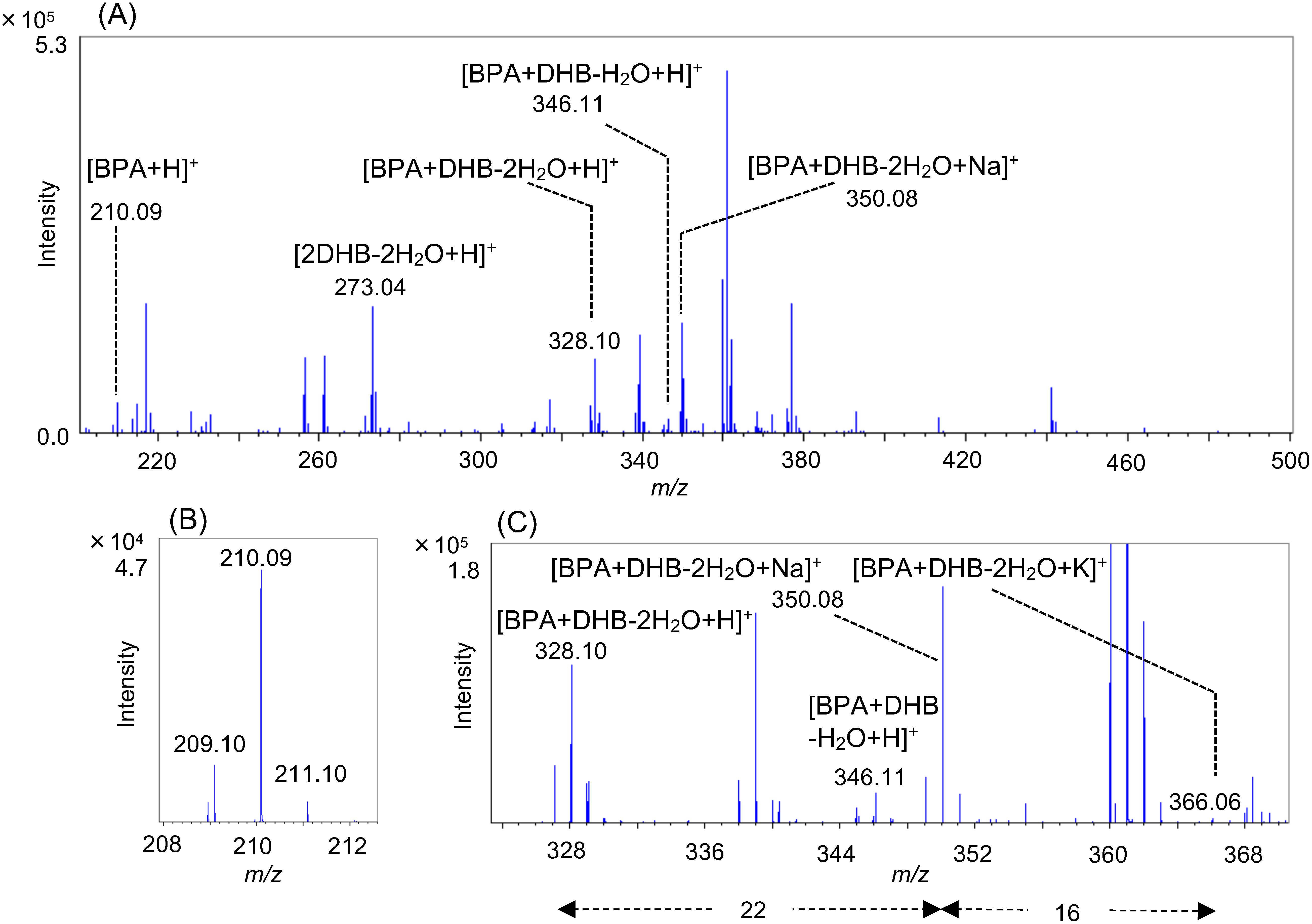
Fig. 2. (A) Mass spectrum of BPA standard (250 pmol) with matrix DHB (B) enlarged spectra of [BPA+H]^+^ and (C) the other BPA ion species. Several dehydrated BPA–DHB complexes were detected similar to dehydrated DHB dimer, [2DHB−2H_2_O+H]^+^. BPA ion species including dehydrated BPA–DHB complexes had the boron isotope ions as same as [BPA+H]^+^. The spectrum data files are available in J-STAGE Data. https://doi.org/10.50893/data.massspectrometry.21523023

The chemical structures of BPA and matrix DHB are shown in Fig. 1. Boron has a unique isotope pattern, and the calculated protonated BPA molecule at *m*/*z* 210.093 ([BPA+H]^+^) and its isotope ions are also shown in Fig. 1. Before MSI measurement, we performed MALDI-MS measurement of BPA standard spot with matrix DHB to confirm the detection of BPA ion species. Figure 2 shows the mass spectrum of 250 pmol-BPA standard spot with DHB. The protonated BPA molecule, [BPA+H]^+^ at *m*/*z* 210.09 was observed with boron isotope ions at *m*/*z* 209 and *m*/*z* 211 as shown in Fig. 2(B). Several ions at *m*/*z* 328, *m*/*z* 346, *m*/*z* 350 and *m*/*z* 366 had isotope ions similar to [BPA+H]^+^ as shown in Fig. 2(C) and these *m*/*z* values were in good agreement with the *m*/*z* values of [BPA+DHB−2H_2_O+H]^+^, [BPA+DHB−H_2_O+H]^+^, [BPA+DHB−2H_2_O+Na]^+^, and [BPA+DHB−2H_2_O+K]^+^, respectively. A small amount of sodium or potassium which is contained in chemical reagents, organic solvent, and laboratory glassware, possibly form the sodium or potassium adduct molecules.

Janda *et al.* reported that several analyte–DHB complexes, phosphocholine, leucine, folic acid, *etc.* were found in the mass spectra obtained from multiple MSI measurements.^28)^ Table 1 lists the measured *m*/*z* values of BPA ion species detected in BPA standard. The differences of the measured *m*/*z* values of BPA ion species from the calculated values were within 1.2 mDa. And the differences of the measured isotope abundance ratios of [BPA+DHB−2H_2_O+H]^+^, [BPA+DHB−2H_2_O+Na]^+^, and [BPA+DHB−2H_2_O+K]^+^ from the calculated ratios were less than 3% as listed in Table 2. Supplementary Fig. 1 shows the product ion spectra obtained from the precursor ions, [2DHB−2H_2_O+H]^+^ at *m*/*z* 273, [BPA+DHB−2H_2_O+H]^+^ at *m*/*z* 328, [BPA+DHB−2H_2_O+Na]^+^ at *m*/*z* 350 and [BPA+DHB−2H_2_O+K]^+^ at *m*/*z* 366 in the MS/MS measurements of BPA standard. A product ion at neutral loss of 136 for each selected precursor ion was observed for above four spectra. Moreover, a product ion at neutral loss of 191 for each selected precursor ion, [BPA+DHB−2H_2_O+H]^+^ and [BPA+DHB−2H_2_O+K]^+^ was observed for each product ion spectrum. Janda *et al.* also reported that the neutral loss of C_7_H_4_O_3_ (136), which matched the molecular formula of matrix DHB without one H_2_O molecule (DHB−H_2_O) from the precursor ion of DHB complex with phosphocholine, PC (36 : 1) was observed in a MS/MS measurement of a brain tissue section.^28)^ The neutral loss of 191 is possibly C_9_H_10_BNO_3_ which matched the molecular formula of BPA without one H_2_O molecule (BPA−H_2_O). Product ions at *m*/*z* 23, *m*/*z* 136 and *m*/*z* 214 obtained from precursor ion, [BPA+DHB−2H_2_O+Na]^+^ were in good agreement with sodium ion, [DHB−H_2_O]^+^ and [BPA−H_2_O+Na]^+^ . Product ions at *m*/*z* 39, *m*/*z* 136, *m*/*z* 175 and *m*/*z* 230 obtained from precursor ion, [BPA+DHB−2H_2_O+K]^+^ matched to potassium ion, [DHB−H_2_O]^+^, [DHB−H_2_O+K]^+^ and [BPA−H_2_O+K]^+^. These results can support the assignments of DHB complexes with BPA, [BPA+DHB−2H_2_O+Na]^+^ and [BPA+DHB−2H_2_O+K]^+^. They are possibly complexes of (DHB−H_2_O) and (BPA−H_2_O). Both DHB and BPA have one phenyl group and three hydroxyl groups. It was thought that hydroxyl group-rich compounds could be favorable to form dehydrated ions, DHB−H_2_O or BPA−H_2_O and phenyl group could promote the cluster formation.

**Table 1:** Table 1. Mass accuracy of BPA ion species detected in BPA standard spot with DHB.

	Calculated *m*/*z*	Measured *m*/*z*	Measured mass error (ppm)
[BPA+H]^+^	210.093	210.0934	0.2
[BPA+Na]^+^	232.075	232.0756	1.3
[BPA+K]^+^	248.049	248.0495	0.9
[BPA+DHB−2H_2_O+H]^+^	328.099	328.0993	1
[BPA+DHB−H_2_O+H]^+^	346.110	346.1101	1.4
[BPA+DHB−2H_2_O+Na]^+^	350.081	350.0806	2.6
[BPA+DHB−2H_2_O+K]^+^	366.055	366.0554	1.4
[BPA+2DHB−2H_2_O+H]^+^	464.115	464.1157	1.2
[BPA+2DHB−H_2_O+H]^+^	482.126	482.1269	2.4

**Table 2:** Table 2. Isotope ion abundance ratio of BPA ion species detected in BPA standard spot with DHB.

	Calculated		Measured
	*m*/*z*	Isotope abundance ratios (%)	Isotope abundance ratios (%)
[BPA+H]^+^	209.097	24	25
	210.093	100	100
	211.097	10	8
[BPA+DHB−2H_2_O+Na]^+^	349.084	24	21
	350.081	100	100
	351.084	18	16
[BPA+DHB−2H_2_O+K]^+^	365.058	24	23
	366.055	100	100
	367.058	20	19

We also examined the detection of BPA standard with matrix CHCA. The intensity of [BPA+H]^+^ with CHCA was approximately 1/5 of that with DHB, although there was no multiple ion formation, such as BPA-CHCA complexes. Therefore, matrix DHB was adopted for BPA mass imaging in brain section.

### MALDI-MSI experiments on a rat brain section with melanoma administered with BPA

**Figure 3:**
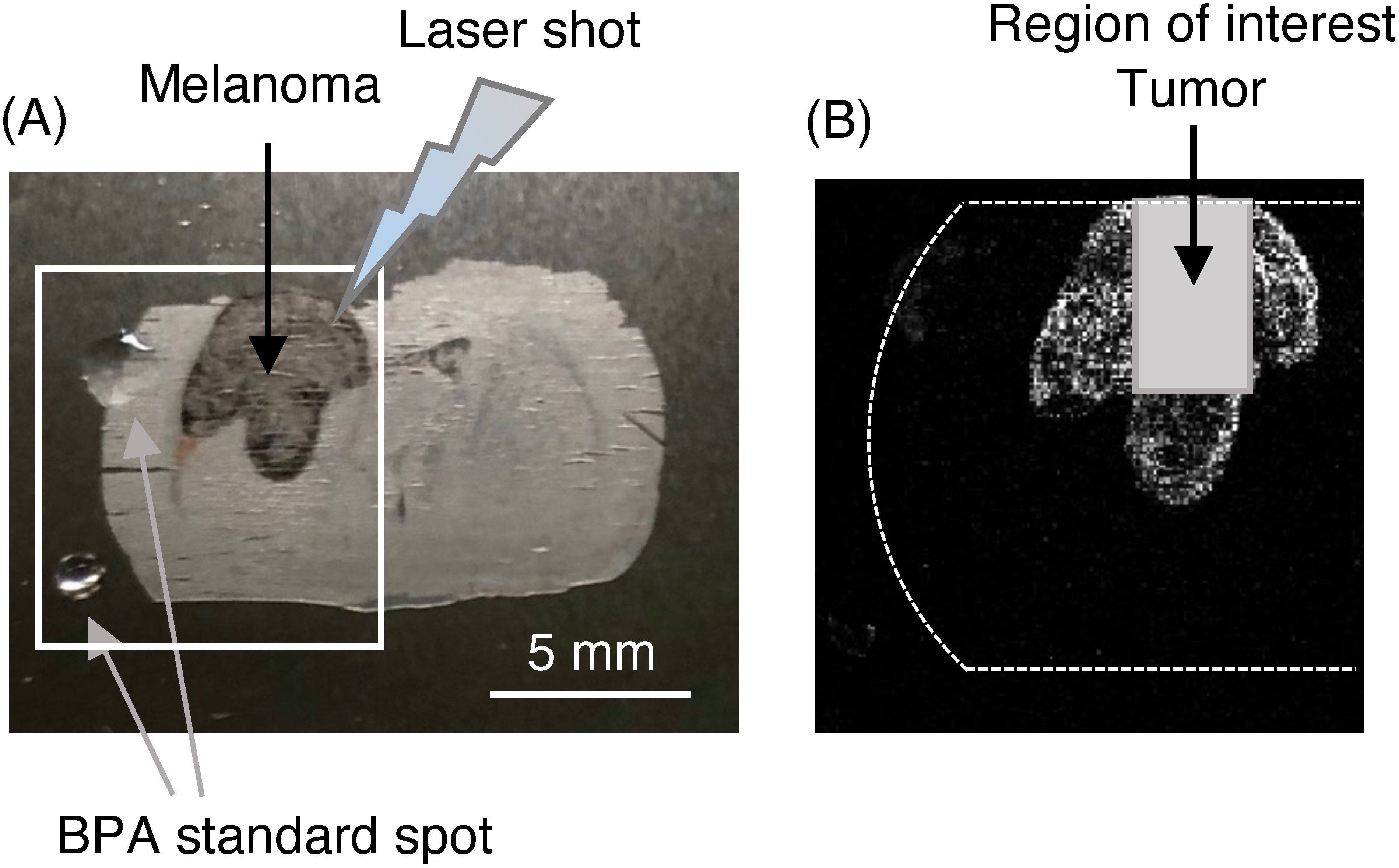
Fig. 3. (A) Brain tissue section with melanoma administered with BPA. MALDI-MSI was performed in the half area enclosed by square. The spotted BPA standard (upper: 100 pmol, lower: 20 pmol) outside brain section were used to confirm the detection of BPA (B) tumor area designated as a region of interest (ROI).

**Figure 4:**
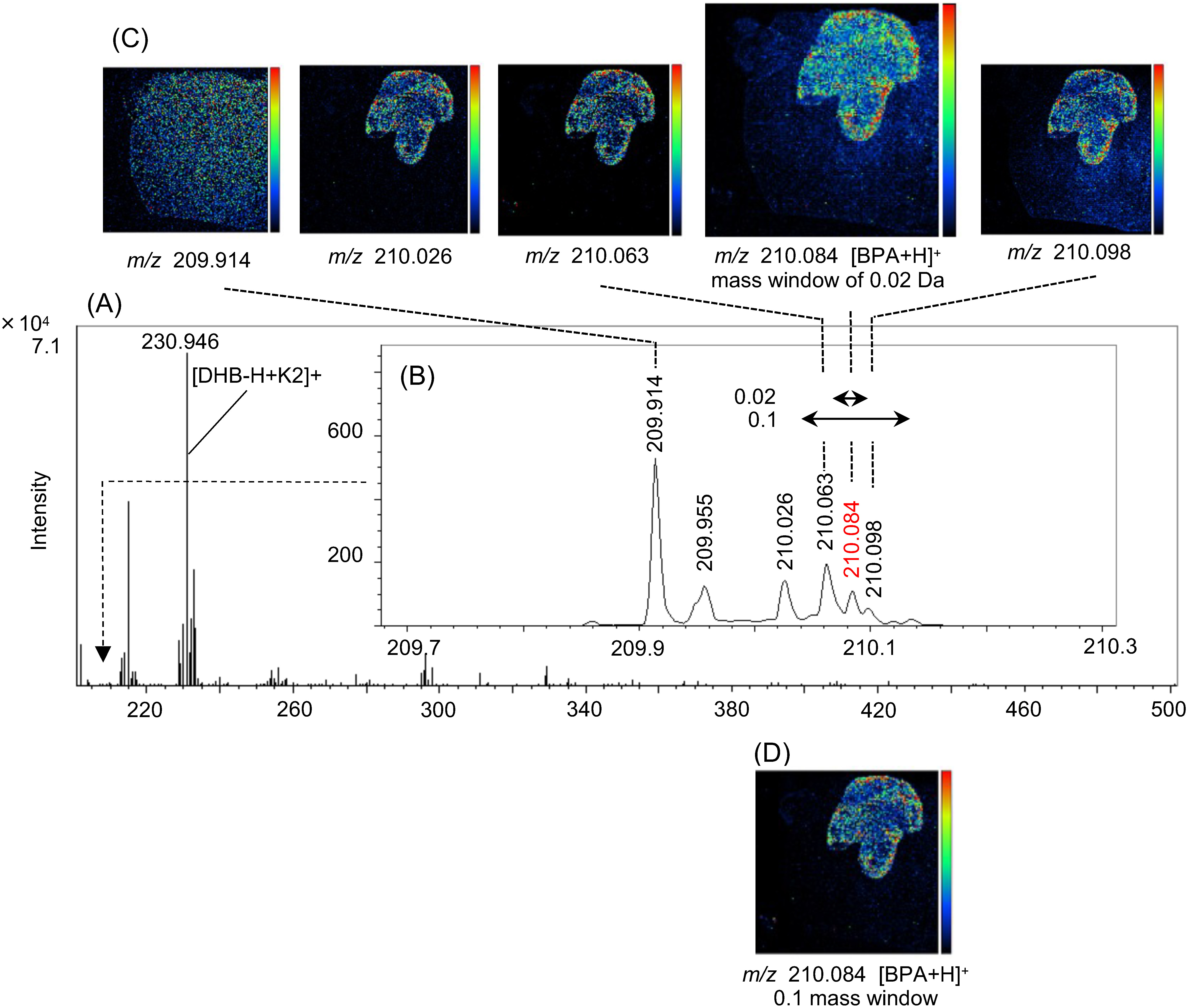
Fig. 4. (A) Averaged mass spectrum per a pixel extracted from tumor region of BPA dosed brain tissue section (B) enlarged mass spectrum around *m*/*z* 210.093 ([BPA+H]^+^) (C) mass images obtained from each peak around *m*/*z* 210 in a mass window of 0.02 Da and (D) mass image obtained from *m*/*z* 210.084 in a mass window of 0.1 Da. The peaks around *m*/*z* 210.093 were separated and each peak width was approximately 0.02 Da. The mass image obtained from *m*/*z* 210.084 in a mass window of 0.02 Da clearly revealed distribution of BPA in tumor region and inside the tumor. The spectrum data files are available in J-STAGE Data. https://doi.org/10.50893/data.massspectrometry.21521988

**Figure 5:**
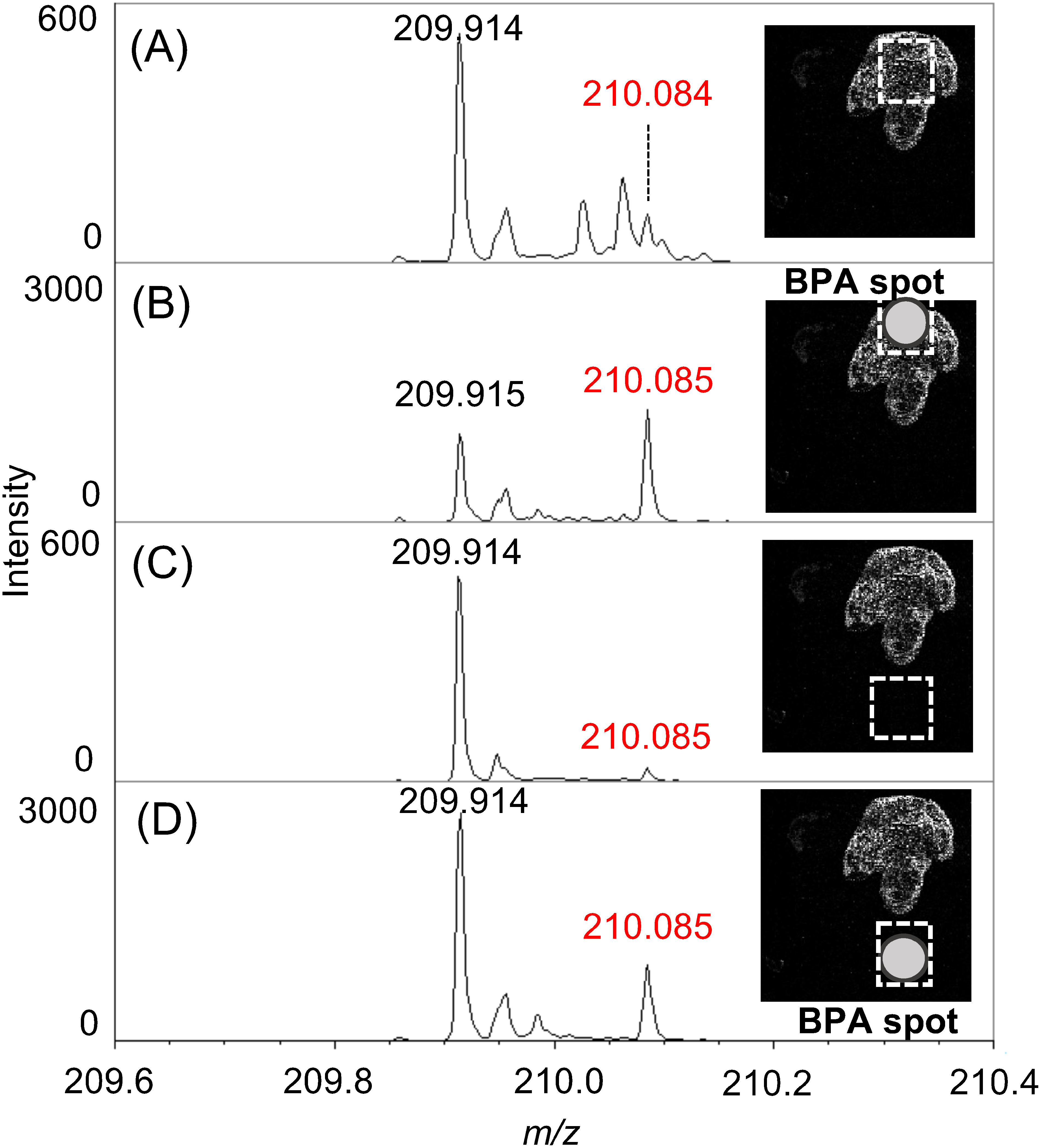
Fig. 5. Averaged mass spectra per a pixel extracted from (A) tumor region, (B) 10 pmol-BPA spotted tumor region, (C) cerebral cortex region and (D) 50 pmol-BPA spotted cerebral cortex region in the BPA dosed brain tissue section. Inserted figures show the position of spotted BPA and the ROI to extract mass spectra. The relative intensities of the peak at *m*/*z* 210.085 to the peak at *m*/*z* 209.9 increased after BPA spotting in tumor or cerebral cortex area.

Figure 3 shows the brain tissue section with melanoma administered with BPA and the location of ROI designated as a tumor region. MSI data acquisitions were performed for the half area of brain section with melanoma. Figure 4 shows the averaged mass spectrum by a pixel extracted from the region of tumor. Also, the enlarged mass spectrum around *m*/*z* 210.093 ([BPA+H]^+^) and the mass images corresponding to each peak around *m*/*z* 210.093 in mass windows of 0.02 Da or 0.1 Da were shown on the top or bottom in Fig. 4. The mass resolution of each peak around *m*/*z* 210.093 was more than 20,000 (FWHM) and their peak width were approximately 0.02 Da. The mass images of the peaks around *m*/*z* 210.093 revealed the shape of tumor. However, the mass image of the peak at *m*/*z* 210.084 in a mass window of 0.02 Da showed the distribution inside the tumor which was different from other peaks at *m*/*z* 210.026, *m*/*z* 210.063 and *m*/*z* 210.098 in a mass window of 0.02 Da, or *m*/*z* 210.084 in a mass window of 0.1 Da. The mass image of the peak at *m*/*z* 209.914 showed the distribution to the whole area of the brain section and it could be assigned to an endogenous biomolecule. To confirm the peak assignment of [BPA+H]^+^ in the sample, the BPA standard was spotted on the tumor or cerebral cortex after MSI measurement and then additional MSI data acquisitions were carried out around the BPA spot area in the tumor or cerebral cortex region. Figure 5 shows the mass spectra before/after the BPA standard spotting on the tumor region (10 pmol) or cerebral cortex region (50 pmol). The relative intensity of *m*/*z* 210.085 to *m*/*z* 209.915 increased in the averaged mass spectrum obtained from the BPA-spotted tumor region (Fig. 5B). The increase in the relative peak intensity at *m*/*z* 210.085 to *m*/*z* 209.914 was also observed in the averaged mass spectrum obtained from the BPA-spotted cerebral cortex region (Fig. 5D). These results suggested that the peak at *m*/*z* 210.084 was assigned to [BPA+H]^+^ in the sample. The mass difference (−9 mDa off) from the calculated value was due to the slight overlap with adjacent peaks, which were originated from biomolecules or matrix DHB. The mass resolution of 20,000 (FWHM) around *m*/*z* 210 was effective to separate peaks around [BPA+H]^+^ in the sample and to identify the peak of [BPA+H]^+^. Therefore, it is highly possible to obtain mass image less overlapping with biomolecules or matrix DHB. However, the difference in color between tumor region and cerebral cortex region in the mass image obtained from [BPA+H]^+^ should be carefully considered because the color contrast in mass image is based on the peak intensity in each pixel which is probably affected by the ionization efficiency of BPA in tumor or cerebral cortex. Actually, the BPA standard spotting experiment suggested the difference of ionization efficiency of BPA in tumor from that in cerebral cortex. Therefore, in order to estimate the parameter T/N ratio using the color contrast in a mass image, the contrast in the mass image obtained from [BPA+H]^+^ should be corrected based on the differences of ionization efficiency which is determined by the BPA standard spotting experiments or experiments combining MSI measurement and quantification of BPA by LC-MS in a same sample.

**Figure 6:**
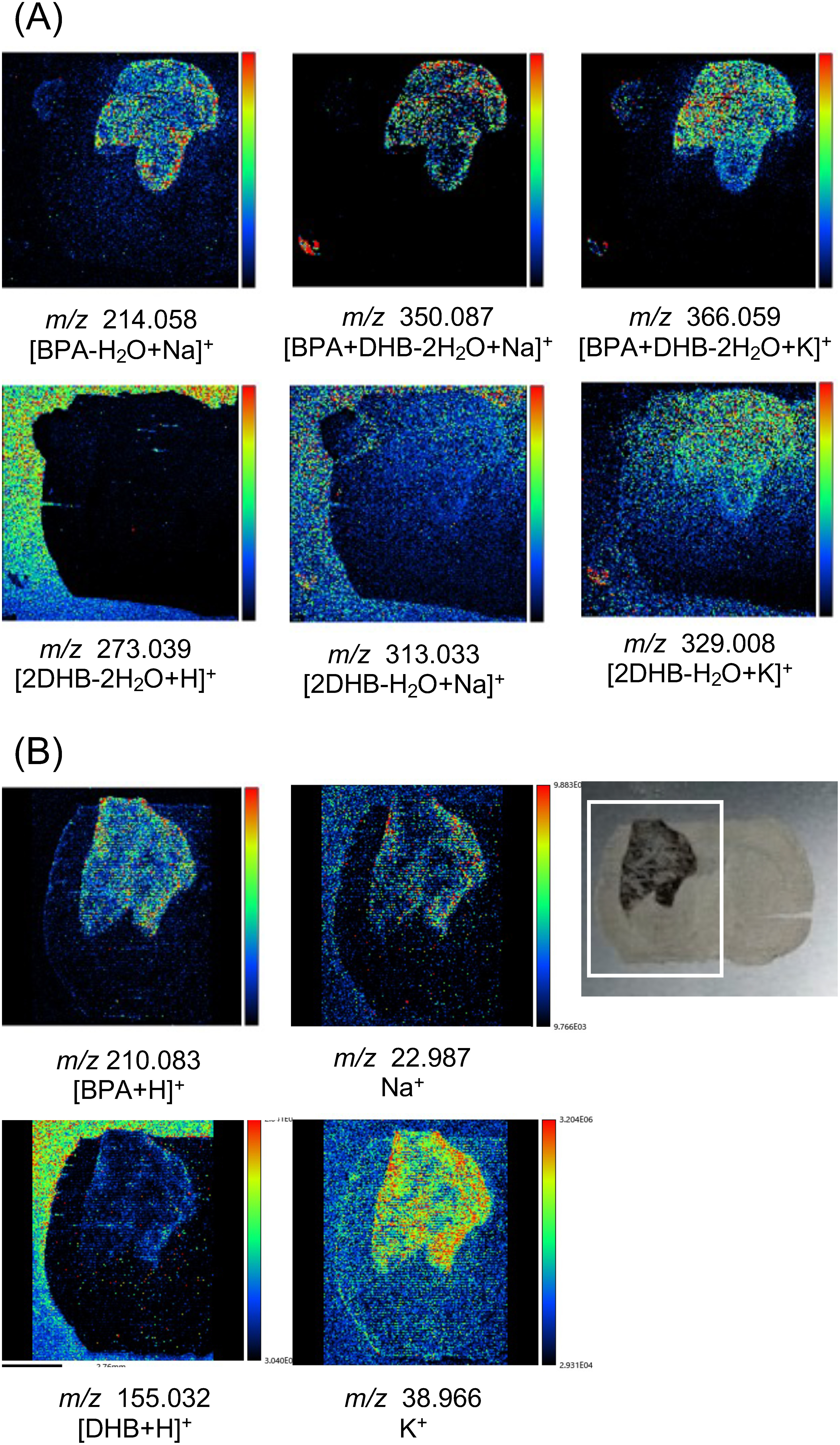
Fig. 6. Mass images obtained from (A) dehydrated BPA–DHB complexes and dehydrated DHB dimers detected in MALDI-MSI measurement with mass range *m*/*z* 205–550 (B) [BPA+H]^+^, [DHB+H]^+^, Na^+^, and K^+^ in MALDI-MSI measurement with mass range *m*/*z* 20–215 and the brain section used in this measurement. MALDI-MSI was performed in the half area enclosed by square. The mass images obtained at *m*/*z* 214.058, *m*/*z* 350.087, *m*/*z* 366.059 revealed the distribution in the tumor region. The mass image of [2DHB−H_2_O+K]^+^ showed clear distribution in tumor region compared to the mass images of [2DHB−H_2_O+Na]^+^ and [2DHB−2H_2_O+H]^+^. The mass image of potassium ion clearly showed the distribution in tumor region and localization inside tumor.

BPA ion species at *m*/*z* 214, 350, or 366 corresponding to [BPA−H_2_O+Na]^+^, [BPA+DHB−2H_2_O+Na]^+^ and [BPA+DHB−2H_2_O+K]^+^ were also detected. The assignments of these peaks were confirmed by BPA-spotting experiment. The supplementary Fig. 2 shows the averaged mass spectra by a pixel around [BPA+DHB−2H_2_O+K]^+^ in the BPA-spotting experiment. The observed *m*/*z* values of BPA species detected in the brain tissue administered with BPA and the observed *m*/*z* values of BPA-spotting experiments were less than 9 mDa (mass error <43 ppm) as shown in Table 3. The mass images obtained from [BPA−H_2_O+Na]^+^, [BPA+DHB−2H_2_O+Na]^+^ and [BPA+DHB−2H_2_O+K]^+^ were shown in Fig. 6(A). And the mass images obtained from dehydrated DHB ion species detected in the same mass range were also shown in Fig. 6(A). The mass image of [2DHB−H_2_O+K]^+^ seemed like similar localization in the tumor region to the mass image of [BPA+DHB−2H_2_O+K]^+^, although the localization in the brain section were not clear in the mass image of [2DHB−2H_2_O+H]^+^ and [2DHB−H_2_O+Na]^+^. We also carried out another MSI measurement in the low mass range at *m*/*z* 20–215 using another brain section administered with BPA to investigate the distribution of matrix DHB, sodium ion and potassium ion in the brain section. The mass images obtained from [BPA+H]^+^, [DHB+H]^+^, sodium ion and potassium ion were shown in Fig. 6(B). The mass image of potassium ion clearly revealed the distribution in the tumor region and also partly localization inside the tumor. In contrast, the distribution in the tumor region and the localization inside the tumor of [DHB+H]^+^ or sodium ion were not clear compared with potassium ion. As the result, the mass image obtained from [BPA+H]^+^ can visualize the distribution of BPA in brain tissue with tumor and also likely showed the localization of BPA inside the tumor. The mass images obtained from [BPA−H_2_O+Na]^+^ and [BPA+DHB−2H_2_O+Na]^+^ might be used to validate the mass image obtained from [BPA+H]^+^. Such validation improve the reliability of results when the sample is a glioma or other cancer in which the boundary between tumor tissue and normal tissue is unclear. However, the mass image of [BPA+DHB−2H_2_O+K]^+^ should be used to estimate the presence of BPA in brain tissue which can be used for the estimation of uptake or excretion of BPA in tumor, because the mass image of [BPA+DHB−2H_2_O+K]^+^ possibly depends on the potassium distribution.

**Table 3:** Table 3. Mass accuracy of BPA ion species detected in the rat brain tissue section administered with BPA in MALDI-MSI measurement.

	Observed values on tumor		Observed values on BPA spotted tumor		Observed values on BPA spotted cortex		Calculated *m*/*z*
	*m*/*z*	Δm (Da)	*m*/*z*	Δm (Da)	*m*/*z*	Δm (Da)	
BPA+H	210.084	−0.009	210.085	−0.008	210.085	−0.008	210.093
BPA−H_2_O+Na	214.058	−0.007	214.058	−0.007	214.058	−0.007	214.065
BPA+DHB−2H_2_O+Na	350.087	0.006	350.081	0	350.081	0	350.081
BPA+DHB−2H_2_O+K	366.059	0.004	366.056	0.001	366.055	0	366.055

## CONCLUSION

We developed an effective method of MALDI-MSI with high mass resolution targeting to [BPA+H]^+^ for BNCT drug, BPA in a brain tumor model rat. The peak of [BPA+H]^+^ at *m*/*z* 210.084 was detected separate from other peaks originated from biomolecules or matrix DHB in the brain sample administered with BPA by achieving mass resolution of approximately 20,000 (FWHM) at *m*/*z* 210. The mass image obtained from [BPA+H]^+^ revealed the distribution in the tumor region in the brain tissue section and also likely showed the localization inside the tumor. The mass image obtained from BPA ion species including [BPA−H_2_O+Na]^+^, [BPA+DHB−2H_2_O+Na]^+^ and [BPA+DHB−2H_2_O+K]^+^ illustrated the distribution in tumor region in the brain tissue section and they could be used to validate the mass image obtained from [BPA+H]^+^ or estimatie the time profile of BPA uptake or excretion. This MSI method has a great potential to evaluate the effect of therapy and damage to normal tissue with near-cellular spatial resolution in BNCT. The color contrast in tumor against cerebral cortex in mass image was probably affected by the difference of BPA ionization efficiency in each tissue. Takeo and Shimma^29)^ reported quantitative mass spectrometry imaging (qMSI) combined with the quantitation using LC-MS/MS. In our next investigation, a method to correct the contrast in mass image including qMSI technique will be developed in order to estimate the T/N ratio in a brain tissue.

## Data Availability

The spectrum data files of Figs. 2, 4, and Supplementary Fig. 1A–1D are available in J-STAGE Data.

## Notes

Mass Spectrom (Tokyo) 2022; 11(1): A0105
