# Supplementary material for: Matrix-Assisted Laser Desorption/Ionization (MALDI) Mass Spectrometry Imaging of L-4-Phenylalanineboronic Acid (BPA) in a Brain Tumor Model Rat for Boron Neutron Capture Therapy (BNCT)
Source: Mass Spectrom (Tokyo). 2022 Dec 20;11(1):A0105. doi: 10.5702/massspectrometry.A0105 (PMC9853116; doi:10.5702/massspectrometry.A0105)
Supplement: Supplementary Data [file massspectrometry-11-1-A0105_s001.pdf]

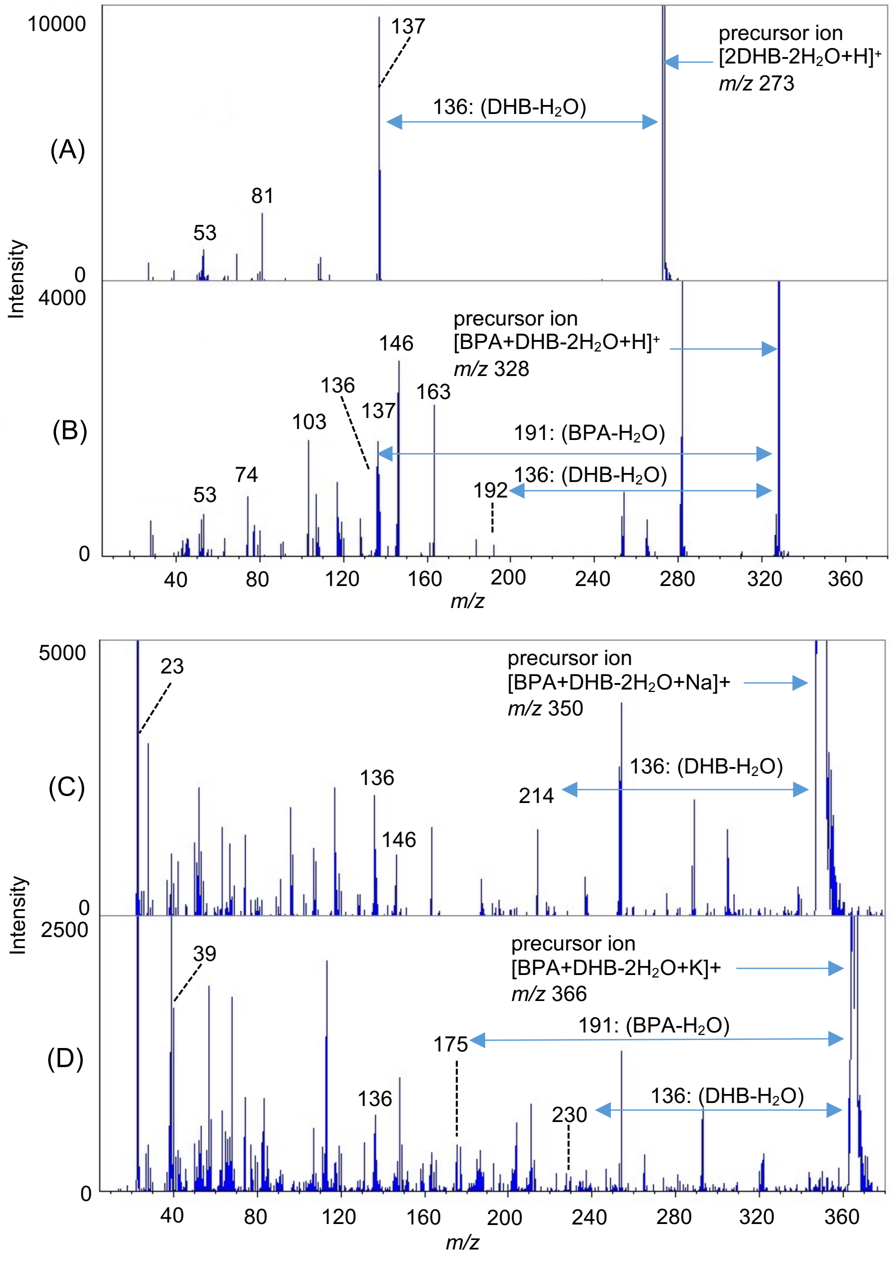

Supplementary figure 1. Product ion spectra obtained from the precursor ions, (A) $[2DHB{-}2H_2O{+}H]^+$ at *m/z* 273, (B) $[BPA{+}DHB{-}2H_2O{+}H]^+$ at *m/z* 328, (C) $[BPA{+}DHB{-}2H_2O{+}Na]^+$ at *m/z* 350 and (D) $[BPA{+}DHB{-}2H_2O{+}K]^+$ at *m/z* 366 in the MS/MS measurement of 250 pmol-BPA standard spot. The neutral loss corresponding to the formula of $(DHB{-}H_2O)$ or $(BPA{-}H_2O)$ was detected in each product ion spectra.
The spectrum data files are available in J-STAGE Data.
https://doi.org/10.50893/data.massspectrometry.21522303

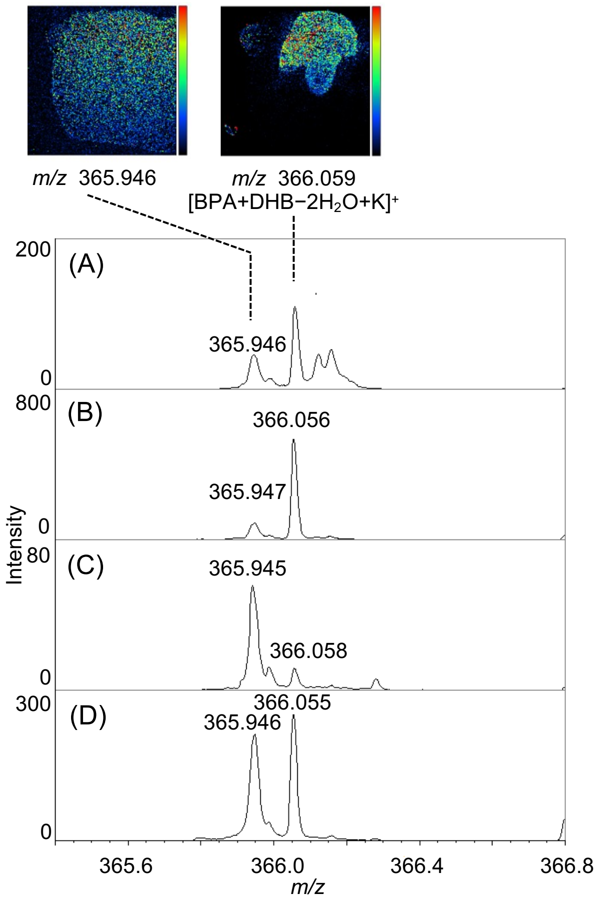

Supplementary figure 2. Mass images obtained from each peak around *m/z* 366 in a mass window of 0.02 Da and the averaged mass spectra by a pixel extracted from (A) tumor region, (B) 10pmol-BPA spotted tumor region, (C) cortex region and (D) 50pmol-BPA spotted cortex region in the BPA dosed brain tissue section. The relative intensities of the peak at *m/z* 366.059 (in the case of A) to the peak at *m/z* 365.95 increased after BPA spotting in tumor or cerebral cortex area.
